# Editorial: Systematic Observation: Engaging Researchers in the Study of Daily Life as It Is Lived

**DOI:** 10.3389/fpsyg.2019.00864

**Published:** 2019-04-24

**Authors:** M. Teresa Anguera, Angel Blanco-Villaseñor, Gudberg K. Jonsson, José Luis Losada, Mariona Portell

**Affiliations:** ^1^Faculty of Psychology, Institute of Neurosciences, University of Barcelona, Barcelona, Spain; ^2^Faculty of Psychology, University of Barcelona, Barcelona, Spain; ^3^University of Iceland, Reykjavik, Iceland; ^4^Department of Psychobiology and Methodology of Health Sciences, Faculty of Psychology, Universitat Autònoma de Barcelona, Cerdanyola del Vallès, Spain

**Keywords:** systematic observation, methodological developments in systematic observation, areas of application in systematic observation, observational design, advances in systematic observation

The Research Topic *Systematic observation: Engaging researchers in the study of daily life as it is lived* (Section *Quantitative Psychology and Measurement*) faithfully reflects the interest of many researchers to conduct studies based on a methodology that is essentially characterized by being highly flexible and rigorous, and that aims to capture reality as it happens when studying it scientifically.

The analysis of a part of reality is complex, given the multifaceted nature of any aspect of daily life. This complexity is manifested in numerous aspects to be considered, from the initial filtering that must be done to conveniently delimit the part of reality to be studied, to the structuring of the ideal observational design, the construction of a customized instrument that allows to properly channel all the behaviors/elements to be observed around the axes or dimensions around which the studied reality pivots, the materialization of a suitably coded record, the management of the records, data quality control, its subsequent analysis, and the interpretation of the results.

This process is none other than the scientific method, although adapted to the reality of natural situations, in which it is not possible or convenient to apply the control that other methodologies offer, given that the spontaneity of behavior and the habituality of the context are of primary concern. In a schematic way, we could say that in the systematic observation the face and cross of a same coin are focused: “Face” because its proven versatility and adaptability make it extremely interesting and demanded in innumerable situations, and “cross” because the rigor of the own scientific method gives it a very estimable value that prestige such studies, as shown in the scientific community. The fact that *Frontiers in Psychology*, a prestigious scientific journal of high visibility around the world, had accepted the proposal of this Research Topic, gives more body and arguments in favor of *systematic observation* to the scientific community.

The 23 articles that make up the Research Topic *Systematic observation: Engaging researchers in the study of daily life as it is lived* are organized from a substantive point of view in different criteria, although each of the published articles could have been “classified” from several points of view.

## Methodological Developments

Given the incessant development of systematic observation over the last quarter of a century, this Research Topic has been an occasion to complete aspects that required progress, in order to be taken as a point of reference in future studies and new developments.

Basically, we differentiate four different aspects that mark a procedural path, and we consider that they are the following:

On the one hand, the conceptualization, development and analysis possibilities of indirect observation (Anguera et al.), which is strongly emerging in recent years, and revealing a wide field of application. More and more researchers are obtaining texts, either by transcribing oral conduct, or by applying new forms of communication, or by direct writing, and they are provided with a procedure to follow.

The weak current of systematic observation has traditionally been the psychometric, and the article by Chacón-Moscoso et al. focuses, in an applied study, on the measurement of quality of observational studies based on content validity, and taking advantage of the possibilities of the Osterlind index.

One aspect that to date had been absent in systematic observation studies is simulation. Manolov and Losada, offer a computer application developed for this purpose, adaptable to different sampling techniques.

And we can also consider as methodological development the work of Izquierdo and Anguera, centered on the notational development of movement, and for which a structured system of rules and symbols is proposed.

## Areas of Application

The studies that we publish in this section stand out for both substantive and procedural aspects within systematic observation, but we have considered that the emphasis that they represent at the level of application areas was the most important:

***a) Sport***

This field presents special characteristics that make systematic observation extremely suitable and attractive as the scientific procedure to be followed.

At the same time, priority is given to the interest in professional football in most sport articles published in this Research Topic, although it is important to take into account specific aspects of each one. While in Zurutuza et al. the objective is oriented to an analysis by physiological variables and is intended to study the relationship of external and internal training load indicators with the objective and subjective fatigue experienced by semi-professional football players, it has a different purpose in other studies. In Diana et al. the interest lies in knowing how game-location positively affects the secondary and tertiary level of performance, prioritizing the incidence of playing at home, or in an opponent field, or in a neutral field. Casal et al., on the contrary, studied the identification of factors that may allow predicting success in professional football, focusing specifically on ball possession, and using bivariate and multivariate statistical analysis.

The orientation is very different in the works of Castañer et al. and Maneiro and Amatria. In both the aim is the in-depth study of elite football players, from the intensive perspective. The analysis of polar coordinates is used in both, and in that of Castañer et al., in addition, the detection of T-Patterns, which was also used in the Diana et al. article.

Morillo et al. address the study of referees in handball, also taking advantage of the extraordinary possibilities of systematic observation. As in the articles by Castañer et al. and Maneiro and Amatria, they apply polar coordinate analysis.

With the exception of the Zurutuza et al. article, which requires complementing the observation with physiological variables, all the other studies mentioned here constructed a custom observation instrument.

***b) Health Psychology***

From a broad definition of Health Psychology, three articles were published in the Research Topic.

On one hand, Sanduvete-Chaves et al. have built a scale that allows measuring the quality of the work climate in emergency services, in which there is usually tension due to the responsibility involved in making decisions and the necessary quickness that is required. In addition to indirect observation, we have worked with questionnaires and surveys.

Arias-Pujol and Anguera is a study of clinical psychology in which the interaction between adolescents in a group therapy has been observed. By means of a polar coordinates analysis it has been possible to analyze the conversation in the therapeutic group, starting from a detailed record obtained by means of a customized observation instrument.

Finally, in Cerezo et al. the influence of parental gender (father/mother) is studied in the interaction with children, and also taking into account the gender of the child; we highlight the study of interaction from the framework of nonlinear dynamic systems.

***c) Educational Psychology***

Systematic observation also has innumerable advantages in studies that revolve around school and learning. In the Research Topic, five articles were published with very different objectives, but with many common elements regarding the procedure.

On the one hand, Rodríguez-Dorta and Borges set out to study the good practices of teachers who attend students with special educational needs. In Escolano-Pérez et al. the systematic observation is complemented with the selective methodology to evaluate the executive functions of preschoolers and analyze their association with later academic skills, using the general linear model from the analytical perspective. Suárez et al. focuses on the teaching of reading by primary school teachers, and, as in Rodríguez-Dorta and Borges and Escolano-Pérez et al., the theory of generalizability is used. The study realized by García-Fariña et al. uses indirect observation, and focuses on the detection of patterns in the verbal behavior of physical education teachers in the school. The article by Santoyo and Mendoza focuses on the study of coercive patterns in the school context, focusing in particular on the description of stability and change in the behavioral patterns of children identified as victims of bullying.

In the five articles of this group, an *ad hoc* observation instrument was elaborated, although in the work of Santoyo and Mendoza it had been presented in a previous study. In Rodríguez-Dorta and Borges, in García-Fariña et al., and in Santoyo and Mendoza, the analytical technique of lag sequential analysis was used, and in Suárez et al. the T-pattern detection and analysis was used, as in the articles by Castañer et al., Diana et al., Diana et al., and Pic Aguilar et al.

***d) Social Psychology***

Taking the term Social Psychology in a broad sense, we refer to the Research Topic articles of this group, which are very diverse among themselves.

Diana et al. focus on the study of deception in social interaction; they combine systematic observation with an experiment, and the T-Patterns detection is applied, as in the articles by Castañer et al., Diana et al., Pic Aguilar et al., and Suárez et al.

Cabrera et al. focus on the objective of studying antisocial behavior, which begins in childhood, remains in adolescence, and continues its escalation during adulthood; this paper explores the social interaction patterns of adolescents, with and without risk of committing antisocial behaviors and over 2 years, in a situation of conversational negotiation about conflicting topics.

Pérez-Tejera et al. is a study of Environmental Psychology that focuses on the study of gender differences in the occupation of public parks; the EXOdES instrument was elaborated, and 35,000 co-occurrences of codes were recorded, with the analysis of polar coordinates being used, as in the articles by Castañer et al., Morillo et al., and Maneiro and Amatria.

***e) Motor Game and Gaze Direction***

Two articles make up this last group:

On the one hand Pic Aguilar et al. focus on the study of motor games, and specifically in triadic ones, in order to know the regularities that are detected from the observable behaviors. The analysis technique used is the detection of T-Patterns, as in the article by Castañer et al., Diana et al. and Diana et al.

And, on the other, Lappi et al. present as objective an expert driver's gaze behavior in natural driving on a real road, without any instruction; and gaze directionality sequences are obtained in the directionality of the gaze.

## Conclusions

In short, the articles included in the Research Topic make up a broad spectrum.

As Editors of this Research Topic, we want to express the satisfaction that comes from having the opportunity to offer the materialization of new studies in the exciting field of systematic observation to the scientific community.

The Research Topic proposal has been motivating, exciting and satisfying, as well as the highest level of acceptance of the originals. Regarding the management, the originals of the 23 articles that make up this Research Topic were sent from December 2016 to November 2017 and were published between April 2017 and November 2018. The time elapsed from submission to publication has ranged between 3 and 21 months (some were delayed due to the difficulty of finding specialist reviewers in the subject), and 100% of the submitted manuscripts were accepted. Now, the average number of views is 3,100.

## Author Contributions

All authors listed have made a substantial, direct and intellectual contribution to the work, and approved it for publication.

### Conflict of Interest Statement

The authors declare that the research was conducted in the absence of any commercial or financial relationships that could be construed as a potential conflict of interest.

